# GLP-1 Receptor Activation Abrogates β-Cell Dysfunction by PKA Cα-Mediated Degradation of Thioredoxin Interacting Protein

**DOI:** 10.3389/fphar.2019.01230

**Published:** 2019-10-25

**Authors:** Shijun He, Wenyu Wu, Yihong Wan, Kutty Selva Nandakumar, Xiuchao Cai, Xiaodong Tang, Shuwen Liu, Xingang Yao

**Affiliations:** ^1^State Key Laboratory of Organ Failure Research, Guangdong Provincial Key Laboratory of New Drug Screening, School of Pharmaceutical Sciences, Southern Medical University, Guangzhou, China; ^2^State Key Laboratory of Ophthalmology, Zhongshan Ophthalmic Center, Sun Yat-sen University, Guangzhou, China; ^3^Center of Pharmacy, Nanhai Hospital, Southern Medical University, Foshan, China; ^4^Center of Clinical Pharmacy, Nanfang Hospital, Southern Medical University, Guangzhou, China

**Keywords:** exendin 4, PKA, Pancreatic beta cells, er stress, beta cell dysfunction

## Abstract

Glucagon-like peptide 1 receptor (GLP-1R) agonist (Exendin-4) is a well-known agent used to improve β-cell dysfunctions *via* protein kinase A (PKA), but the detailed downstream molecular mechanisms are still elusive. We have now found that PKA Cα mediated- TXNIP phosphorylation and degradation played a vital role in the β-cell protective role of exendin-4. After PKA activator (Exendin-4 or FSK) treatment, PKA Cα could directly interact with TXNIP by bimolecular fluorescence complementation and Co-IP assays in INS-1 cells. And PKA Cα overexpression decreased TXNIP level, whereas TXNIP level was largely increased in PKA Cα-KO β-cells by CRISPR-Cas9. Interestingly, TXNIP overexpression or PKA Cα-KO has impaired β-cell functions, including loss of insulin secretion and activation of inflammation. PKA Cα directly phosphorylated TXNIP at Ser307 and Ser308 positions, leading to its degradation *via* activation of cellular proteasome pathway. Consistent with this observation, TXNIP (S307/308A) mutant resisted the degradation effects of PKA Cα. However, exendin-4 neither affected TXNIP level in TXNIP (S307/308A) mutant overexpressed β-cells nor in PKA Cα-KO β-cells. Moreover, exendin-4 treatment reduced the inflammation gene expression in TXNIP overexpressed β-cells, but exendin-4 treatment has no effect on the inflammation gene expression in TXNIP (S307/308A) overexpressed β-cells. In conclusion, our study reveals the integral role of PKA Cα/TXNIP signaling in pancreatic β-cells and suggests that PKA Cα-mediated TXNIP degradation is vital in β-cell protective effects of exendin-4.

## Introduction

The prevalence of type 2 diabetes (T2D) in human beings has been increasing over recent several decades (Gao et al., 2016; Zheng et al., 2018). Endoplasmic reticulum (ER) stress is a major underlying cause of T2D, indicated by increasing evidence, and following pancreatic β-cell dysfunctions (Laybutt et al., 2007; Shang et al., 2014). Indeed, efficient ER functions plays an important role in β-cells function because of the large demand for insulin synthesis and secretion (Rutter et al., 2015). As pro-insulin is processed to mature insulin in ER, the enhancement of insulin production puts a stress on ER functions and leads to the activation of ER stress and the unfolded protein response in the pancreatic β-cells. Furthermore, many factors could also cause or aggravate ER stress in β-cells, such as inflammatory cytokines and free fatty acids (Zhou et al., 2012; Lu et al., 2013). Eventually pancreatic islets reveal symbols of ER stress from mice and humans with T2D (Montane et al., 2014). Recent studies indicate β-cell inflammasome activation induced by ER stress, and following IL-1β production, which could cause a feedback loop to enlarge inflammatory responses, finally leading to cell damage (Szpigel et al., 2018).

Previous reports suggests ER stress and inflammation act as a relevant function in β-cells dysfunctions or death (Donath, 2014). Recent studies indicated that thioredoxin-interacting protein (TXNIP) is the crosslink between ER stress and inflammatory effects in pancreatic β-cells (Oslowski et al., 2012). TXNIP was a TRX-binding protein that regulated the antioxidant functions of TRX, which maintained redox balance in the cells and had a protective role against oxidative stress mainly by scavenging reactive oxygen species, though its functions seem to be more varied (Huang et al., 2016). TXNIP overexpression could inhibit cell proliferation through inhibition of cell cycle. In addition, TXNIP- knockout get cancer more easily than the normal mice, and also with hematopoiesis problems. Moreover, TXNIP^–/–^ mice show impaired glucose homeostasis. A recent study also indicated that TXNIP directly interacted with NLRP3 and induced IL-1β secretion in β-cells (Zhou et al., 2010). Combined with these phenomena that ER stress could induce TXNIP overexpression by PERK or IRE1 pathways, IL-1β mRNA transcription is then enhanced and its maturation is induced by the NLRP3 activation, thus, resulting β-cell death is mediated by ER stress (Oslowski et al., 2012). However, it is unclear whether there exists a mechanism or drug type to regulate TXNIP degradation in β-cell.

Exendin-4, the anti-diabetic drug, could improve β-cell dysfunctions and apoptosis in a PKA-dependent manner under ER stress condition (Cunha et al., 2009; Yusta et al., 2006). PKA is a serine/threonine kinase formed by a dimer of two regulatory (R) subunits and two catalytic (C) subunits, but the holoenzyme is inactive because the R subunits bind with C subunits and only the C subunits have the ability to phosphorylate PKA substrates after disassociation with R subunit (Lester et al., 1997). There are four types of R subunits (RIα, RIβ, RIIα, and RIIβ) and two types of C subunits (C-α and C-β). RIα or RIβ subunits construct the type I PKA, and RIIα or RIIβ subunits construct the Type II PKA holoenzymes. Type I PKA is activated at a lower cAMP level compared with type II PKA. In β-cells, type I and type II PKA could be detected (Shibasaki et al., 2014). Interestingly, the acute phase of insulin secretion was increased and blood glucose was effective control in PKA Cα subunit knock in mice (Kaihara et al., 2013), so PKA Cα was overexpressed or knocked out throughout this study. Also, a previous study demonstrated that exendin-4 or forskolin (FSK) treatment could largely decrease the high glucose-induced TXNIP level in pancreatic β-cells (Shao et al., 2010). These observations raise the possibility that PKA could mediate TXNIP level under ER stress condition in response to exendin-4 treatment.

Considering ER stress could directly activate inflammation through TXNIP, whether ER stress-activated inflammation could be modulated by exendin-4 is still unclear. Here, we found that activation of PKA has largely reduced TXNIP level and inflammation under ER stress condition. Moreover, PKA Cα could directly interact with TXNIP and lead to its degradation. PKA Cα/TXNIP signaling was thus found to lead to a vital function in the effects of exendin-4. In conclusion, our study was an exploratory study and provided a missing link between GLP-1R, PKA Cα and TXNIP. 

## Materials and Methods

### Experimental Materials

Thapsigargin (THAP, T9033) was purchased from Sigma–Aldrich Company with a purity >98% (HPLC). H89 (S1643, purity 98%) and forskolin (FSK, S1612, purity 99%) were from Biotime Company. Exendin-4 (HY-13443) was from MedChem Express Company with a purity 98.96%. RPIM1640 medium (C11875500BT), DMEM medium (C11995500BT) FBS (10099141), and supplements were from Thermo Fisher, MTT (97062-376, purity 98%) and puromycin (10mg/ml, AAJ67236-8EQ) was purchased from VWR. DAPI (KGR0001-1) was from Keygen Biotech. Polybrene Transfection Reagent (TR-1003-G) was purchased from EMD Millipore Corporation. pBiFC-VC155 (P0681) and pBiFC-VN173 (P0680) were from Miaolingbio Company.

### Cell Culture

INS-1 832/13 cells (INS-1 cells) were a gift from Professor Yong Liu and grown in RPMI 1640 medium as previously reported (Yao et al., 2015; Lu et al., 2016a). All experiments were performed with this culture unless otherwise specified.

### Plasmids Construction and Transfection

PKA Cα (Gene ID: 25636) was inserted into both pEYFP-C1 vector at SalI and BamHI site and pBiFC-VC155 vector at EcoRI and KpnI site. TXNIP (Gene ID: 117514) was inserted into both pECFP-N1 vector at NheI and HindIII site and pcDNA3.1(+)-Myc-His A vector at HindIII and BamHI site. TXNIP (S308A) or TXNIP (S307/308A) mutated genes were inserted into pBiFC-VN173 vector at HindIII and XbaI site and pcDNA3.1(+)-Myc-His A vector at HindIII and BamHI site. Lipo2000 (ThermoFisher, CA, USA) were used for transient transfections for western blotting, confocal and Co-IP assay.

### MTT Assay

INS-1 cells were cultured in 96-well plates with normal cell culture overnight at a density of 2 × 10^4^ cells/well. These cells were then treated with FSK or exendin-4 and THAP (0.5 µM) for 24 h, following 3-(4,5-dimethylthiazol-2-yl)-2,5-diphenyltetrazolium bromide (MTT, 0.5 mg/ml) incubation for 4 h. DMSO (200 µL/well) was added into the well to resolve the formazan crystals and the absorbance was detected using a Tecan microplate reader at 570 nm (Tecan, Mannedorf, Switzerland).

### Glucose-Stimulated Insulin Secretion (GSIS) Assay

GSIS assay was performed as our previous report (Zhang et al., 2013; Yao et al., 2015). Briefly speaking, INS-1 cells were pre–incubated with KRB buffer contained 0.2% BSA for 2 h, followed by incubation in KRB buffer containing glucose (16.8 mM) with indicated compounds for 2 h. Then, the supernatant was collected and insulin concentration was detected by the Insulin High Range Kit (Cisbio, Billerica, France).

### Co-Immunoprecipitation (Co-IP) Assay

INS-1 cells were plated into 6-well plates and then incubated in 5% CO_2_ for 48 h for Co-IP assay incubated with normal cell culture. IP was performed by the Classic IP kit (ThermoFisher, CA, USA). The cell (1 mg of protein) was lysed with lyse buffer (25 mM Tris, 150 mM NaCl, 1 mM EDTA, 1% NP-40, 5% glycerol, pH 7.0) and was used for IP with anti-TXNIP or anti- PKA Cα antibodies at 4°C for 12 h. Proteins were then centrifuged and pellets were washed 3 times with cold TNTE 0.1% (50 mM Tris–HCl buffer pH 7.4, 150 mM NaCl, 5 mM EDTA and 0.1% Triton X-100). Pellets were re-suspended in Laemmli buffer (100 mM Tris–HCl pH6.8, 20% glycerol, 2% SDS, 0.05% bromophenol blue and 100 mM DTT) and boiled for 5 min. The proteins were analyzed by western blotting assay.

### Western Blotting (WB) Assay

Antibodies against TXNIP (14715), PKA Cα (5842) and β-actin (4970) were purchased from Cell Signaling Technology. For WB assay, INS-1 cells were cultured into plates (6-well), and then cell lysate was separated by SDS–PAGE and transferred into PVDF membranes. All membranes were visualized using FluorChem E (Protein simple, CA, USA) after incubation with indicated antibodies for 12 h. The image densitometry was analyzed by ImageJ 1.48 software.

### qRT-PCR Assay

Quantitative reverse transcription-PCR (qRT-PCR) experiments were performed using LightCycler480 (Roche Diagnostics International, Rotkreuz, Switzerland) using GoTaq^®^ qPCR Master Mix Kit (Promega Corporation, WI, USA) and PrimeScript^™^RT Master Mix Kit (Takala Bio Inc, Kusatsu, Japan). Total RNA was isolated using Cell Total RNA Isolation Kit (Foregene, Sichuan, China). The concentration and the quality of RNA were measured using DS-11 FX Spectrophotometer (DeNovix Inc, DE, USA). Primers used for the qRT-PCR are given below:

GAPDH Forward: 5’-TGACCTCAACTACATGGTCTACA-3’,
GAPDH Reverse: 5’-CTTCCCATTCTCGGCCTTG-3’,
IL-1β Forward: 5’-GTGTGGATCCCAAACAATACCC-3’,
IL-1β Reverse: 5’-AAGACAGGTCTGTGCTCTGC-3’,
TXNIP Forward: 5’-CAAGTTCGGCTTTGAGCTTC-3’
TXNIP Reverse: 5’-ACGATCGAGAAAAGCCTTCA-3’,
IL-6 Forward: 5’-CCAACTCATCTTGAAAGCACTTGAAG-3’,
IL-6 Reverse: 5’-CATTCATATTGCCAGTTCTTCGTAGAG-3’.

### Bimolecular Fluorescence Complementation (BiFC) Assay

The basic principle of the BiFC assay is to fuse two non-fluorescent fragments from a split fluorescent Venus protein to two interaction partners. If the two proteins interact with each other, the interaction brings the two non-fluorescent fragments together to allow reconstitution of an intact fluorescent protein molecule. Here, pBiFC-VN173 and pBiFC-VC155 were used. PKA Cα and TXNIP were fused into these plasmids separately. Then these plasmids were transfected into INS-1 cells for 24 h. After being fixed with 3.7% formaldehyde in PBS and stained with DAPI, the cells were visualized using a 100x objective lens to observe the BiFC signal (Ex/Em: 515/528 nm).

### CRISPR-Cas9 Mediated PKA Cα Knockout

INS-1cells were transfected with the lentiCRISPRv2 [lentiCRISPRv2 puro was a gift from Brett Stringer (Addgene plasmid # 98290)] with the guide RNA sequence 5’-CCTCCCAATCCGCCGTAAGT-3’ against PKA Cα (Gene ID: 25636). These plasmids were packaged into retroviral particles by co-transfecting into 293T cells with lentiCRISPRv2- PKA Cα construct having plasmids expressing psPAX2 (psPAX2 was a gift from Didier Trono (Addgene plasmid # 12260)) and pCMV-VSV-G (pCMV-VSV-G was a gift from Bob Weinberg (Addgene plasmid # 8454)) (Stewart et al., 2003). Supernatants were collected and used to transduce 10^5^ INS1 cells in the presence of 10 µg/mL polybrene. After 24 h post transduction, cells were selected in 1 µg/mL puromycin.

Puromycin-resistant cells were single-cell sorted into a 96-well plate and colonies were grown up. Cloned INS1 cells were screened for homozygous mutations that disrupted the coding sequence of PKA Cα as follows. 100 cell colonies were used to prepare the whole cellular DNA. The region of the guide RNA was amplified using the following primers:

5’-CTCTCCCTGCTCCCAGAGAA-3’
5’- ATGAGTCCAAGGCCAGCTTC -3’.

Amplified genomic DNA was Sanger sequenced to determine the nature of the CRISPR-CAS9-mediated genomic disruption. A cell line with confirmed homozygous disruption of PKA Cα (**Figure 4A**) was then screened out.

### Statistical Analysis

All of the data are expressed as mean ± SEM. Multiple comparisons were performed by one-way ANOVA analysis followed by Bonferroni post-hoc test (compare all pairs of columns), when variances were equal between groups. All statistical analyses were performed using Prism 5 Graph Pad software. In all cases, if the P value determined from a statistical test was less than 0.05, the difference was considered statistically significant. Values are presented as ns, *P < 0.05, **P < 0.01, and ***P < 0.001. All experiments were performed in triplicate. All statistical testing is exploratory, and not hypothesis-testing.

## Results

### PKA Activation Promoted ER Stress-Induced TXNIP Degradation

**Figure 1:**
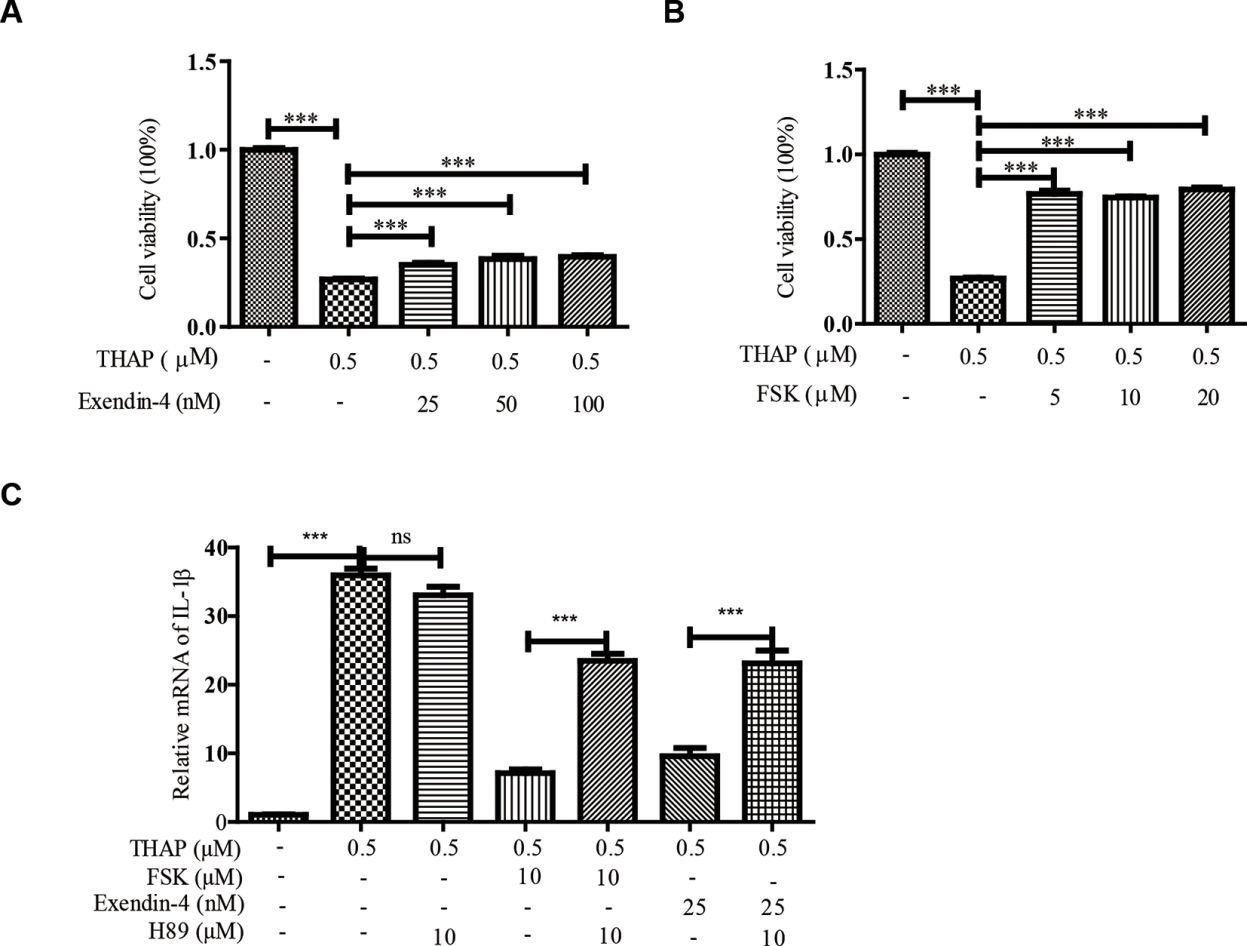
Exendin-4 or FSK treatment reduces ER stress-induced β-cell viability. INS-1 cells were incubated with THAP (0.5 µM), with **(A)** exendin-4 or **(B)** FSK at indicated concentration for 24 h, then the cellular viability was analyzed by MTT assay (n = 5). INS-1 cells were incubated with THAP (0.5 µM), H89, with **(C)** exendin-4 or **(D)** FSK at indicated concentration for 24 h, then the IL-1β level was analyzed by qRT-PCR (n = 3). Bars represent the mean ± SEM of independent samples. Significant difference in expression between un-treated group and the drug treatment group as labeled was analyzed by one-way ANOVA, corrected for multiple comparisons with the Bonferroni test. *** indicates P value < 0.001).

ER stress induces TXNIP expression, which in turn activates inflammasomes and finally β-cell death (Ortsater and Sjoholm, 2007; Oslowski et al., 2012). Whereas, ER stress could be alleviated by exendin-4 (GLP-1 receptor agonist) *via* PKA activation in pancreatic β-cells (Kim et al., 2010). In order to confirm these results, we thus treated INS-1 cells with thapsigargin (THAP), an ER stress inducer, to observe the effect of exendin-4 or FSK on β-cell viability, because exendin-4 or FSK both could activate PKA. Similar to the previous results, exendin-4 (**Figure 1A**) or FSK (**Figure 1B**) treatment could statistically significantly improve ER stress-induced β-cell death. Considering ER stress-induced inflammation is the cause of β-cell death (Oslowski et al., 2012), we evaluated the effects of FSK on IL1-β level. As shown in **Figure 1C**, THAP largely enhanced IL1-β transcription, which was reduced in the presence of exendin-4 or FSK. Therefore, we wanted to know whether the anti-inflammation effect was dependent on PKA. After PKA activation was inhibited by H89, a PKA inhibitor, IL1-β, was at the same level under ER stress condition with or without exendin-4 or FSK treatment. Moreover, H89 could not induce more IL-1β expression under ER stress, which excluded the possibility that the inhibition of PKA has other downstream effects that increase the IL-1β expression. The results indicated that PKA played a key role in the protective effect of exendin-4 or FSK.

**Figure 2:**
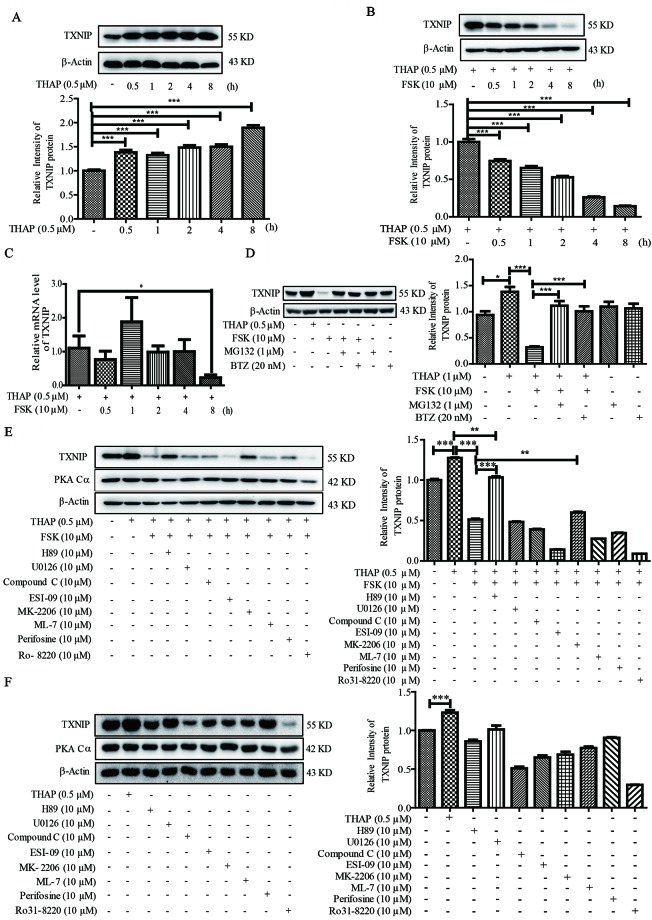
FSK treatment reduces TXNIP level. **(A)** INS-1 cells were incubated with THAP (0.5 µM), and TXNIP protein was detected using WB (n = 3). **(B)** INS-1 cells were incubated with THAP (0.5 µM) and FSK (10 µM) at the same time, and then TXNIP protein was detected using WB (n = 3). **(C)** INS-1 cells were incubated with THAP (0.5 µM) and FSK (10 µM) together, then mRNA level of TXNIP was detected by qRT-PCR (n = 3). **(D)** INS-1 cells were incubated with MG132 (1 µM) or Bortezomib (0.5 µM) for 1 h, and then THAP (0.5 µM) and FSK (10 µM) was added for 2 h, then TXNIP was detected using WB (n = 3). **(E)** INS-1 cells were incubated with indicated inhibitors for 1 h, and then THAP (0.5 µM) and FSK (10 µM) was added for 8 h, then TXNIP and PKA Cα were detected using WB (n = 3). **(F)** INS-1 cells were incubated with indicated inhibitors for 8 h, and then TXNIP and PKA Cα were detected using WB (n = 3). Bars represent the mean ± SEM of independent samples. Significant difference in expression between un-treated group and the drug treatment group as labeled was analyzed by one-way ANOVA, corrected for multiple comparisons with the Bonferroni test. (* indicates P value < 0.05, ** indicates P value < 0.01, *** indicates P value < 0.001).

Considering ER stress-induced TXNIP locates at the upstream of IL1-β, we therefore explored whether PKA activation could regulate TXNIP level under ER stress condition in β-cells. THAP statistically significantly induced TXNIP expression as early as 0.5 h post-treatment, which lasted for 8 h (**Figure 2A**). This observation was consistent with a previous report (Oslowski et al., 2012). However, FSK treatment largely decreased TXNIP protein level induced by ER Stress, as early as 0.5 h (**Figure 2B**). These results encouraged us to find out whether TXNIP transcriptional level was also inhibited by FSK. As shown in **Figure 2C**, FSK (10 µM) had no effect on the mRNA level of TXNIP induced by THAP after 0.5-4 h treatment, except 8 h. Furthermore, it wasfound that FSK reduced TXNIP mRNA level at 12, 24 and 48 h treatment in our lab (data not shown). From the above, these results indicated that FSK mainly promoted TXNIP degradation other than at the transcriptional level at short time incubation.

It is well known that the ubiquitin proteasome plays a key role in proteometabolism in the eukaryotic cells. We thus investigated the role of the proteasome in the progress of FSK-mediated TXNIP degradation. MG132 and bortezomib, two proteasome inhibitors, were used to inhibit the proteasome activity (Kitagaki et al., 2015), which could largely abolish the FSK-mediated TXNIP degradation (**Figure 2D**). Together, these data suggested that the FSK-mediated TXNIP degradation progress was mainly dependent on proteasome-mediated protein degradation system.

Moreover, recent studies suggested that TXNIP could be degraded after phosphorylation by AMPK or AKT (Shaked et al., 2011; Wu et al., 2013; Li et al., 2015; Waldhart et al., 2017; Zaragoza-Campillo and Moran, 2017). To clarify the involvement of the AMPK, AKT or other enzymes activated by FSK in TXNIP degradation process, several inhibitors of these kinases were used. INS-1 cells were pre-incubated with these enzyme inhibitors for 1 h, and were then treated with FSK or THAP for 8 h. MEK1/2 (U0126), CREB (ESI-09), AKT (Perifosine), PKC (Ro 31-8220 and ML-7) and AMPK (Compound C) inhibitors had little effect on the reduction of TXNIP expression by FSK (**Figure 2E**). Treatment with these inhibitors alone could not enhance TXNIP accumulation (**Figure 2F**). On the other hand, AKT inhibitor (MK-2206) reversed the effect of FSK on TXNIP degradation, which accounted for about 14%. However, H89 reversed the effect of FSK on TXNIP degradation about 71%. Indeed, H89 mainly reversed the effect of FSK on TXNIP level (**Figure 2E**). These results suggested a priority role of PKA on TXNIP level.

### PKA Directly Interacted With TXNIP

**Figure 3:**
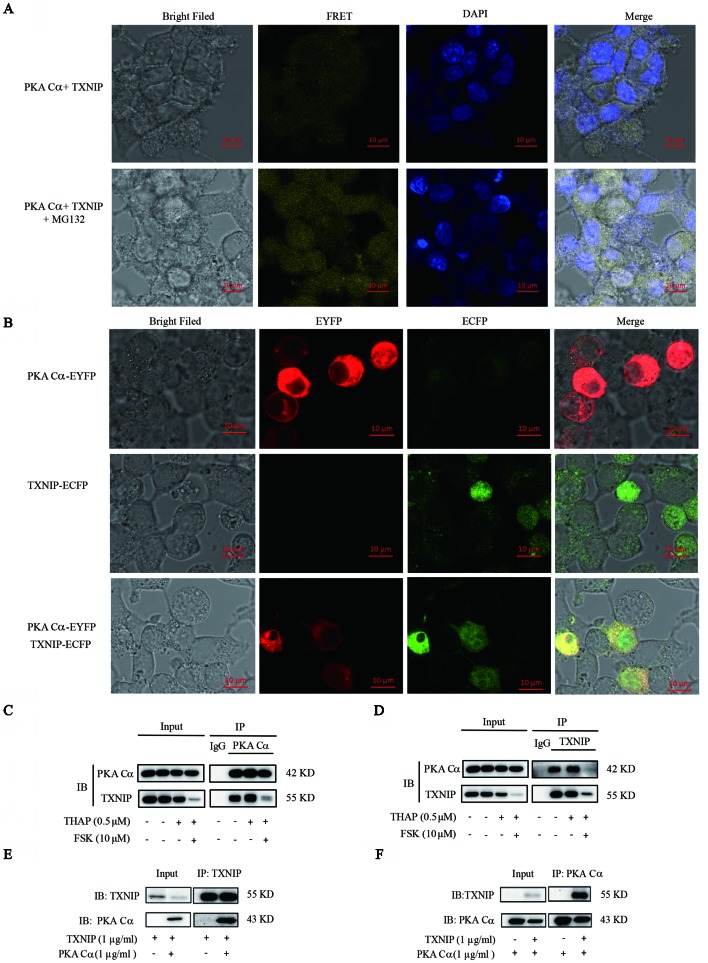
PKA Cα directly interacts with TXNIP. **(A)** TXNIP-VC155 (0.5 µg/ml) and PKA Cα-VN173 (0.5 µg/ml) plasmids were transfected into INS-1 cells, with or without MG132. The FRET signal (Ex/Em: 515/528 nm) was observed using confocal microscope after 24 h. Nucleus was stained with DAPI (n = 3). **(B)** ECFP-TXNIP or EYFP- PKA Cα plasmids were transfected into INS-1 cells separately or together for 24 h. EYFP (red) or ECFP (green) signal was observed using confocal microscope (n = 3). INS-1 cells were incubated with THAP (0.5 µM) or FSK (10 µM) for 2 h, followed by lysis, anti- PKA Cα **(C)** or anti-TXNIP **(D)** antibody was used for Co-IP assay (n = 3). HEK-293 cells were transfected with PKA Cα or TXNIP (3.1a) plasmid and then cells were lysed. PKA Cα **(E)** or TXNIP **(F)** antibody was used for Co-IP assay (n = 3).

**Figure 4:**
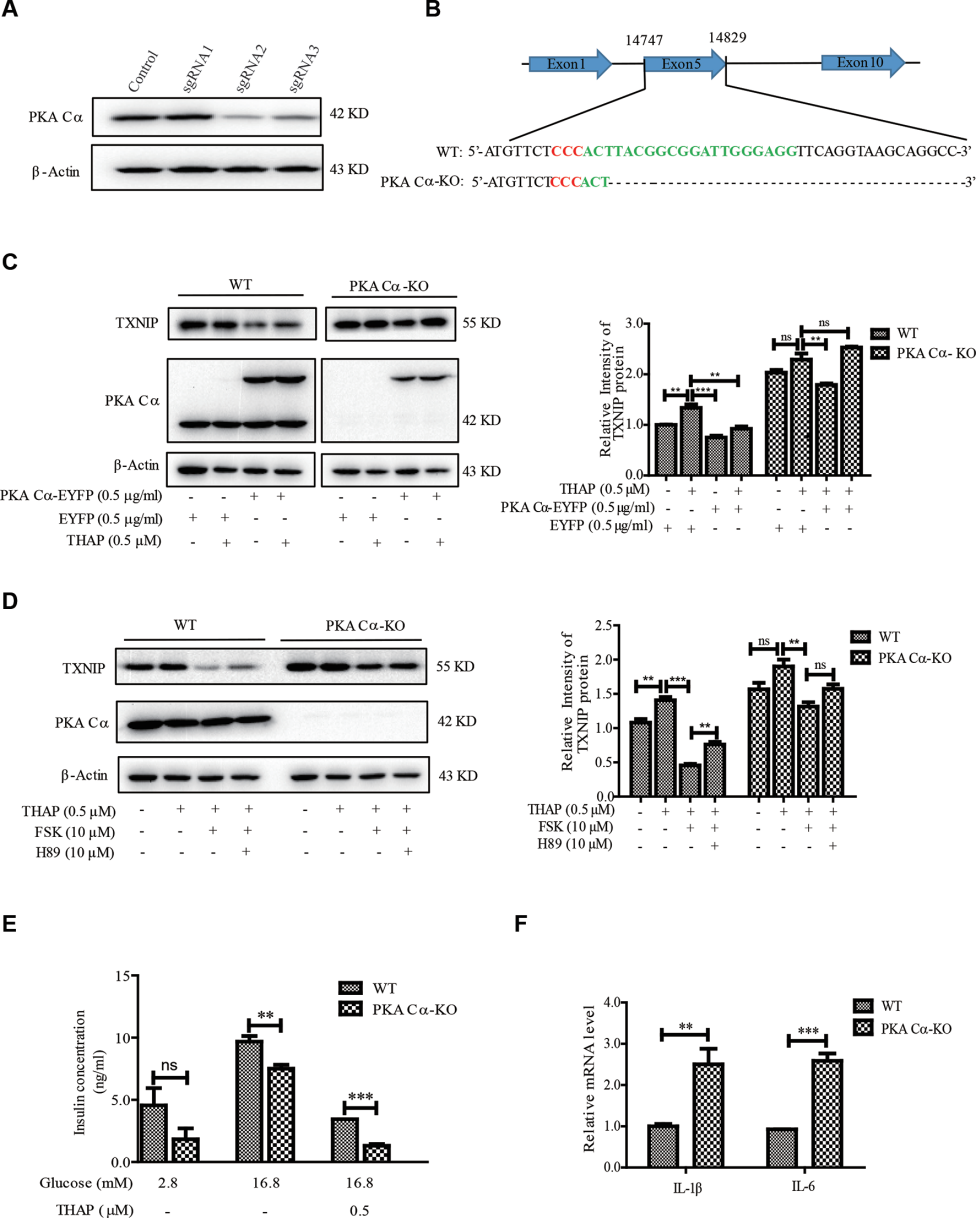
Deletion of PKA Cα enhances TXNIP level in β-cells. **(A)** Three designed sgRNAs were inserted into LentiV2 plasmid. These plasmids were transfected into INS-1 cells, and the TXNIP protein level was detected using WB (n = 3). **(B)** Single PKA Cα-KO β-cell was screened out using 96-well plate and the PKA Cα gene was sequenced in WT and PKA Cα-KO cells. Red sequence indicates GGG in the CRISPR -Cas9 sgRNA design and the green sequence indicates the whole sgRNA sequence. **(C)** EYFP vector or EYFP- PKA Cα plasmids were transfected into WT or PKA Cα-KO INS-1 cells separately for 24 h. THAP was added to the indicated cells. TXNIP and PKA Cα were analyzed using WB (n = 3). **(D)** WT or PKA Cα-KO INS-1 cells were treated with THAP, FSK or H89 for 2 h. TXNIP and PKA Cα were analyzed using WB (n = 3). **(E)** WT or PKA Cα-KO INS-1 cells were treated with THAP, and GSIS assay was performed as described in materials and methods (n = 3). **(F)** The mRNA level of IL-1β and IL-6 were analyzed in WT or PKA Cα-KO INS-1 cells by qRT-PCR (n = 3). Bars represent the mean ± SEM of independent samples. Significant difference in expression between groups as labeled was analyzed by one-way ANOVA, corrected for multiple comparisons with the Bonferroni test. (ns indicates no statistically significant, ** indicates P value < 0.01, *** indicates P value < 0.001).

The above results indicated that there might be a relationship between PKA and TXNIP, but it still lacks of any direct evidence. The existence of direct interactions between these two proteins could be studied using BiFC assay, which has been widely used recently to validate protein-protein interactions (Shyu et al., 2008). Hence, we constructed TXNIP-VC155 and PKA Cα-VN173 plasmids, and these plasmids were transfected into INS-1 cells. The complementation only occurred when TXNIP interacted with PKA Cα, and was not be observed when a VC155 fragment alone was expressed with PKA Cα-VN173, indicating no spontaneous interactions occurred between the two Venus fragments. There were weak fluorescent signals in INS-1 cells with TXNIP-VC155 and PKA Cα-VN173 plasmids transfection (**Figure 3A**), which may because PKA Cα overexpression promoted TXNIP degradation and was confirmed in the flowing result (**Figure 4C**). On the other hand, MG132 was used to inhibit TXNIP degradation by PKA Cα, and there were bright fluorescent signals by TXNIP-VC155/PKA Cα-VN173 interactions, compared with the control group (**Figure 3A**). This phenomena was further confirmed by the above results that PKA Cα-mediated TXNIP degradation was dependent on the proteasome, which also demonstrated that TXNIP directly interacted with PKA Cα.

Furthermore, there was an overlap between TXNIP and PKA Cα position in the cytoplasm. ECFP-tagged TXNIP and EYFP-tagged PKA Cα were used to detect the location of these two proteins in INS-1 cells. As shown in **Figure 3B**, TXNIP (shown in green) and PKA Cα (shown in red) shared the same location (shown in yellow) in the cytoplasm of INS-1 cells.

Apart from these observations, the direct interactions of PKA Cα/TXNIP was further confirmed by Co-IP assay in INS-1 cells. PKA Cα was immunoprecipitated using anti- PKA Cα antibodies, and TXNIP was clearly found to be co-precipitated with PKA Cα (**Figure 3C**). Furthermore, PKA Cα was co-precipitated with TXNIP using anti-TXNIP antibodies as well (**Figure 3D**). To further confirm this phenomena, we transfected TXNIP and PKA Cα expression plasmids into HEK293T cells. As predicted, PKA Cα was markedly co-precipitated by anti-TXNIP antibodies (**Figure 3E**), and *vice versa* (**Figure 3F**). These data provided solid evidence that PKA Cα directly interacted with TXNIP.

### PKA Cα Knockout Increased TXNIP Level and Led to Impair Pancreatic β-Cell Functions

The fact that PKA Cα interacted with TXNIP directly was what prompted us to study the potential regulatory effects of PKA Cα in modulating TXNIP level and pancreatic β-cell dysfunctions. Considering PKA Cα is important for PKA activity in β-cells (Goehring et al., 2007; Shibasaki et al., 2014), we constructed PKA Cα knockout (PKA Cα-KO) cell line for this purpose. Three sgRNAs were designed for PKA Cα knockout using CRISPR-cas9 system and sgRNA2 showed the best knockout efficiency (**Figure 4A**). A single cell clone of PKA Cα-KO was then screened out and sequenced, in which the PKA Cα was found to have a deletion mutation (**Figure 4B**). As expected, there was a high level of TXNIP level in PKA Cα-KO cells compared to the WT cells (**Figure 4C**). This data indicated that TXNIP degradation was blocked after PKA Cα knockout. In addition, the effect of FSK on TXNIP protein level was further evaluated in PKA Cα-KO cells, and THAP could hardly enhanced TXNIP level in PKA Cα-KO cells since cellular TXNIP was accumulated without its degradation by PKA (Figures 4C, D). Moreover, the TXNIP degradation ability of FSK was largely decreased in PKA Cα-KO cells compared with the control cells. On the other hand, H89 could not reverse the effect of FSK on TXNIP level in PKA Cα-KO β-cells (**Figure 4D**). These results were in accordance with the result presented in **Figure 2E**, which revealed an important effect of PKA Cα on TXNIP degradation.

It is widely reported that TXNIP overexpression has direct links with glucotoxicity, insulin secretion, β-cell inflammation and cell death (Chen et al., 2008; Reich et al., 2012). We thus evaluated the ability of glucose-stimulated insulin secretion (GSIS) in PKA Cα-KO cells. It was shown that insulin secretion was statistically significantly down regulated in PKA Cα-KO β-cells compared with WT β-cells in the presence of 16.8 mM glucose (**Figure 4E**). Moreover, insulin concentration reduced about 64.8% and 81% in WT β-cells and PKA Cα-KO β-cells under ER stress condition, respectively (**Figure 4E**). These data indicated that the TXNIP level was enhanced in PKA Cα-KO β-cells, thereby impairing insulin secretion. Furthermore, the inflammation level was evaluated in PKA Cα-KO β-cells. Compared to the WT β-cells, there were statistically significantly enhanced transcriptional levels of pro-inflammatory cytokines, such as IL-1β and IL-6 in PKA Cα-KO β-cells (**Figure 4F**).

Taken together, the above data demonstrated that TXNIP was largely enhanced in PKA Cα-KO β-cells, in which accumulation finally led to pancreatic β-cell dysfunctions.

### PKA Cα Overexpression Reduced TXNIP Level and Improved Pancreatic β-Cell Functions

Considering PKA Cα-knockout could increase TXNIP level and induce pancreatic β-cell dysfunctions, we thus speculated that PKA Cα overexpression could reduce TXNIP level and improve β-cell functions. At first, we transfected PKA Cα-EYFP or EYFP overexpression plasmid into WT or PKA Cα-KO β-cells. PKA Cα overexpression statistically significantly reduced the TXNIP level under normal conditions (**Figure 4C**). This result further confirmed that PKA Cα could promote TXNIP degradation, which was similar to the result shown in **Figure 3A**. Next, we detected the effect of PKA Cα overexpression on TXNIP level under ER stress condition. PKA Cα overexpression could reduce TXNIP level in both WT and PKA Cα-KO β-cells in the presence of THAP (**Figure 4C**). These data indicate that PKA Cα could directly decrease TXNIP level both under normal and ER stress condition.

**Figure 5:**
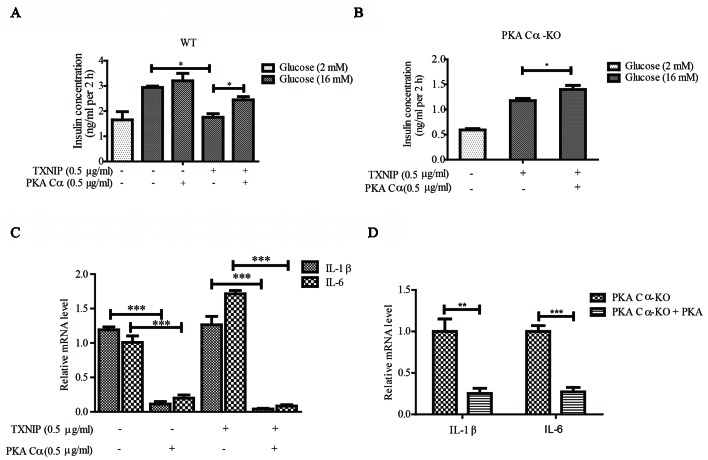
PKA Cα overexpression alleviates TXNIP-induced β-cell dysfunctions. TXNIP and PKA Cα plasmids were transfected into INS-1 cells **(A)** or PKA Cα-KO INS-1 cells **(B)**, and GSIS assay was performed as described in materials and methods (n = 3). **(C)** TXNIP plasmid was transfected into WT or PKA Cα-KO INS-1 cells, with or without EYFP- PKA Cα plasmids. Then the mRNA level of IL-1β and IL-6 were analyzed using qRT-PCR (n = 3). **(D)** The mRNA level of IL-1β and IL-6 were analyzed by qRT-PCR in PKA Cα-KO INS-1 cell, with or without PKA Cα transfection (n = 3). Bars represent the mean ± SEM of independent samples. Significant difference in expression between groups as labeled was analyzed by one-way ANOVA, corrected for multiple comparisons with the Bonferroni test. (* indicates P value < 0.05, ** indicates P value < 0.01, *** indicates P value < 0.001).

Next, we evaluated the effect of PKA Cα overexpression on GSIS under TXNIP overexpression condition. PKA Cα overexpression could not enhance insulin secretion level under normal condition. However, PKA Cα overexpression could reverse the effect of TXNIP overexpression, which largely down regulated the insulin secretion level (**Figure 5A**). In addition, we found that the insulin concentration was reduced to almost half the level in PKA Cα-KO β-cells (1.5 ng/ml), compared to in WT β-cells (3 ng/ml), and this effect was moderately ameliorated by PKA Cα overexpression (**Figure 5B**).

We further evaluated the effects of PKA Cα on TXNIP-induced inflammation in β-cells. As expected, TXNIP overexpression has statistically significantly induced the mRNA level of the pro-inflammatory cytokines, IL-1β and IL-6 (**Figure 5C**). However, these genes induced by TXNIP expression were reduced with PKA Cα overexpression (**Figure 5C**). Moreover, the mRNA level of IL-1β and IL-6 were enhanced in PKA Cα-KO β-cells (**Figure 5D**). However, PKA Cα overexpression abolished the increasing rate of IL-1β and IL-6 mRNA in PKA Cα-KO β-cells, as expected. These data suggested that PKA Cα overexpression could reduce TXNIP level and β-cell inflammation.

### TXNIP (S307/S308) was the Phosphorylated Substrate of PKA Cα

**Figure 6:**
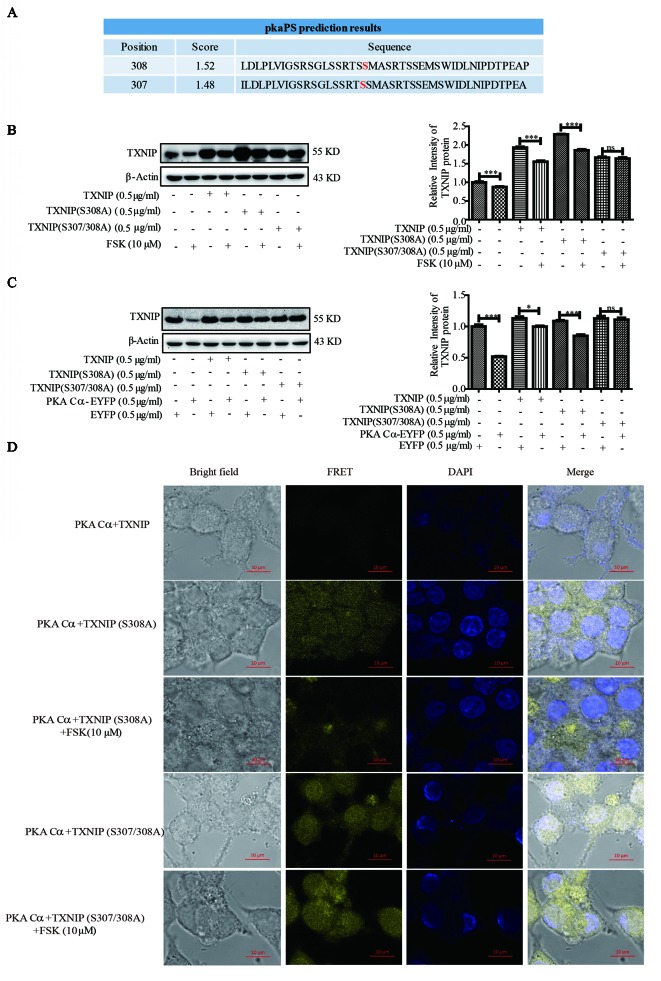
TXNIP (S308A) mutant resists the degradation effects of PKA Cα. **(A)** TXNIP phosphorylation site prediction by pkaPS software (n = 3). **(B)** TXNIP, TXNIP (S308A) and TXNIP (S307/308A) were transfected into INS-1 cells separately, then the TXNIP protein level was detected using WB after FSK treatment for 2 h (n = 3). **(C)** TXNIP, TXNIP (S308A), TXNIP (S307/308A) and EYFP- PKA Cα plasmids were transfected into INS-1 cells for 24 h and TXNIP level was detected using WB (n = 3). **(D)** TXNIP-VC155 (0.5 µg/ml), TXNIP (S307/308A)-VC155 (0.5 µg/ml), TXNIP (S308A)-VC155 (0.5 µg/ml) and PKA Cα-VN173 (0.5 µg/ml) plasmids were transfected separately into INS-1 cells, and the FRET signal (Ex/Em: 515/528 nm) was observed using confocal microscope after 24 h. Nucleus was stained with DAPI (n = 3). Bars represent the mean ± SEM of independent samples. Significant difference in expression between groups as labeled was analyzed by one-way ANOVA, corrected for multiple comparisons with the Bonferroni test. (ns indicates no statistically significant, * indicates P value < 0.05, *** indicates P value < 0.001).

Given that PKA Cα is a well-known phosphokinase, we analyzed whether TXNIP could be phosphorylated by PKA Cα, leading to its degradation. TXNIP is highly conserved among species, with a sequence similarly of 98∼99%. These sequences were analyzed using pkaPS software, a website able to predict S/T residues phosphorylated by PKA (http://mendel.imp.ac.at/sat/pkaPS/) (Neuberger et al., 2007). The predicted top3 phosphorylation sites of TXNIP based on the predicted score was shown in **Figure 6A**. S308 residue received the highest score, followed by S307, S314 (**Figure 6A**), as reported earlier (Shaked et al., 2011; Wu et al., 2013). Therefore, we constructed TXNIP (S308A) and TXNIP (S307/308A) to detect the degradation effects of PKA Cα. TXNIP (WT), TXNIP (S308A) or TXNIP (S307/308A) plasmids were transfected separately into β-cells with or without FSK. TXNIP (S308A) expression showed partial resistance to FSK-mediated degradation (**Figure 6B**). However, TXNIP (S307/308A) expression showed complete resistant ability to FSK-mediated degradation (**Figure 6B**). Moreover, TXNIP (S308/307A) expression could resist PKA Cα-mediated degradation with PKA Cα overexpression (**Figure 6C**). However, Ser314 mutation could not inhibit the PKA-medicated TXNIP degradation (data not shown). These results suggested that S307/S308 residue of TXNIP played a vital role in PKA Cα-mediated phosphorylation.

Furthermore, the above phenomena was confirmed by BiFC assay, TXNIP -VC155, TXNIP (S308A)-VC155, TXNIP (S307/308A)-VC155 or PKA Cα-VN173 plasmids were transfected into INS-1 cells to observe the fluoresce signals (**Figure 6D**). As expected, the fluorescent signal was brighter in TXNIP (S308/307A) overexpressed β-cells than in TXNIP (S308A) overexpressed β-cells. Even after FSK treatment, the fluorescence intensity was not decreased in TXNIP (S307/308A) overexpressed β-cells. These data indicated that TXNIP was phosphorylated by PKA Cα at Ser307 and Ser308 positions.

### PKA Cα/TXNIP Pathway Participated in the Protective Role of Exendin-4

**Figure 7:**
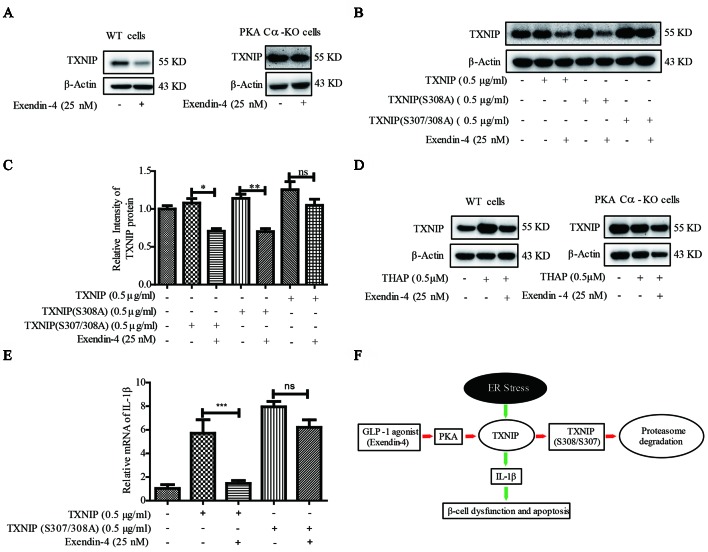
Exendin-4 mediated β-cell protective effects are dependent on PKA Cα/TXNIP pathway. **(B)** **(A)** WT and PKA Cα-KO pancreatic β-cells were treated with exendin-4 for 2 h and TXNIP level was detected using WB (n = 3). **(B)** TXNIP, TXNIP (S308A) and TXNIP (S307/308A) plasmids were transfected separately into INS-1 cells for 24 h, followed by treatment with exendin-4 for 2h. TXNIP level was detected using WB (n = 3). **(C)** The quantification of **(B)**. **(D)** WT and PKA Cα-KO pancreatic β-cells were treated with THAP or exendin-4 for 2 h and TXNIP level was detected using WB (n = 3). **(E)** TXNIP and TXNIP (S307/308A) mutant plasmids were transfected separately into INS-1 cells for 24 h. The mRNA level of IL-1β and IL-6 were detected using qRT-PCR (n = 3). **(F)** The graphic model of PKA Cα/TXNIP pathway in pancreatic β-cells. Red arrow indicated the negative effect, Green arrow indicated the positive effect. Bars represent the mean ± SEM of independent samples. Significant difference in expression between groups as labeled was analyzed by one-way ANOVA, corrected for multiple comparisons with the Bonferroni test. (ns indicates no statistically significant, * indicates P value < 0.05, ** indicates P value < 0.01, *** indicates P value < 0.001).

It was documented earlier that exendin-4 modulates TXNIP (Shao et al., 2010), but the downstream mechanisms are not clear. Also, whether exendin-4 has an effect on TXNIP-medicated β-cell inflammation is far from clear. Exendin-4 is reported to alleviate ER stress-induced β-cell dysfunctions *via* PKA here we investigated the role of PKA Cα/TXNIP pathway in exendin-4 mediated protective effects of β-cells. Similar to an earlier report (Shao et al., 2010), TXNIP level was statistically significantly reduced by exendin-4 treatment. However, there was no change in the level of TXNIP after exendin-4 treatment in PKA Cα-KO β-cells (**Figure 7A**). This data indicated that the effects of exendin-4 on TXNIP was dependent on PKA Cα activation.

Based on our above results using TXNIP (S307/S308) mutant in PKA Cα-mediated TXNIP phosphorylation, we transfected TXNIP (S307/308A) mutant plasmid into β-cells to study the effects of exendin-4. TXNIP protein level was statistically reduced by exendin-4 in β-cells transfected with TXNIP. Unlike TXNIP transfection, TXNIP (S307/308A) mutant has retained the high level of TXNIP with the incubation of exendin-4 (**Figure 7B**). This result further confirmed the role of TXNIP (S307/308A) in a PKA Cα-mediated phosphorylation event.

In addition, we evaluated the effects of exendin-4 on TXNIP under ER stress condition. TXNIP level was enhanced by THAP treatment, but when treated with exendin-4, ER stress-induced TXNIP level was statistically significantly reduced in WT cells (**Figure 7C**). Moreover, THAP has lost its TXNIP enhancement ability in PKA Cα-KO cells, which was similar to the results of **Figure 4C**. This might be ER stress researched saturated state in PKA Cα-KO cells, so THAP could not induce more TXNIP. However, the effects of exendin-4 disappeared in PKA Cα-KO β-cells under similar conditions (**Figure 7C**).

We further evaluated the effects of exendin-4 on inflammation in TXNIP or TXNIP (S307/308A) mutant overexpressed β-cells. Both TXNIP or TXNIP (S307/308A) overexpression up-regulated the IL-1β mRNA level. However, exendin-4 treatment decreased the IL-1β mRNA level in TXNIP overexpressed β-cells (**Figure 7D**), but the effect of exendin-4 was disappeared in TXNIP (S307/308A) mutant overexpressed β-cells (**Figure 7D**). These data clearly demonstrated the role of the PKA Cα/TXNIP pathway in the anti-inflammatory effects of exendin-4 (**Figure 7E**).

## Discussion

Exendin-4 is an effective agonist of the glucagon-like peptide 1-(7-36)-amide receptor of pancreatic β-cells, which could improve β-cell functions, such as insulin secretion, ER stress and ER stress-related apoptosis (Cunha et al., 2009; Shao et al., 2010; Zhang et al., 2014a). However, the detailed downstream mechanism is still not clear. In this study, we demonstrated that exendin-4 or FSK (PKA activator) could alleviate ER stress-induced β-cell dysfunctions through PKA Cα mediated TXNIP degradation pathway. Although earlier studies reported the effects of exendin-4 on TXNIP, our results demonstrated the crucial role of PKA Cα-mediated TXNIP phosphorylation on the β-cell protective effects of exendin-4 for the first time.

The signaling pathways mediated by cAMP are well known, and many biological processes under both physiological and pathological conditions are regulated by cAMP. Previous studies implicated Epac (exchange protein directly activated by cAMP) in exendin-4-cAMP signaling participated in the pancreatic β-cells protection from high-glucose (Shao et al., 2010). Except Epac, exendin-4 or FSK could also activate various proteins through promotion of intracellular cAMP concentrations in β-cells, such as PKA, CaMK, MAPK, ERK1/2, PI3K, AKT, PKC-ζ, AMPK, and. In our study, using specific inhibitors, we found that only PKA Cα participated in the TXNIP degradation effects of FSK under ER stress condition. Our data excluded the role of Epac in the TXNIP degradation effects, which might be due to different pathways operating on β-cells dysfunctions. Moreover, AKT and AMPK had weak TXNIP degradation effects in β-cells, which was contradictory to the previous observations made with liver cells, the reason for which might be the different downstream mechanisms in different types of cells. Although FSK decreased the mRNA level of TXNIP at 8 h treatment, FSK treatment significantly decreased TXNIP protein level as early as 0.5 h (**Figure 2B**). Based on these results, we tended to think that FSK mainly reduced TXNIP at protein level other than affecting transcriptional level at short time incubation. Moreover, we think that the long time incubation effect of FSK may be due to the CREB, which enters into nuclear to regulate gene expression once activated by PKA. Collectively, we think PKA activation promoted TXNIP degradation at the short time incubation, while eventually inhibiting the TXNIP transcriptional level for a long time.

Given TXNIP as a substrate of AMPK and AKT in hepatic cells (Shaked et al., 2011; Wu et al., 2013; Waldhart et al., 2017), we wanted to know whether TXNIP could be directly modulated by PKA Cα in pancreatic β-cells. At first, BiFC assay was used to detect TXNIP interactions with PKA Cα. The intracellular interactions between TXNIP and PKA Cα brought the two non-fluorescent fragments into a close position to make the reconstitution of an intact fluorescent protein molecule. The bright fluoresce signaling observed has strongly confirmed the interactions between TXNIP and PKA Cα. This result was further confirmed after using Co-IP and confocal assays. To the best of our knowledge, this is the first report to observe the direct interactions between TXNIP and PKA Cα in β-cells.

PKA, a well-known serine/threonine protein kinase, regulates various cellular activities by phosphorylating specific targets in the cells that contain a sequence pattern (RRX(S/T)YY)) in the amino acid residues (Smith and Scott, 2018; Soberg et al., 2017). We have now found a RXXS (308) sequence in TXNIP, which might be phosphorylated by PKA Cα. TXNIP phosphorylation residues were also predicted by a kinase binding model, which gave similar results to Ser308, having the highest score for phosphorylation activity. Although a lack of an antibody against phosphorylated TXNIP is an issue, the upward shift of TXNIP observed after FSK treatment in IP assay was same as in a previous report (Wu et al., 2013; Waldhart et al., 2017). Conversely, TXNIP (S307/308A) mutant showed resistance to PKA Cα activation-mediated degradation. Our results clearly demonstrated that TXNIP (Ser307/308) was phosphorylated by PKA Cα but not by AMPK and AKT kinases.

In cells with high glucose levels and in cells from diabetic patients, TXNIP is up regulated, which then induces β-cell inflammation and apoptosis (Zhang et al., 2014;Lu et al., 2016b). It has been proposed that TXNIP inhibition could be a novel approach for β-cell death protection, β-cell mass promotion and diabetes prevention (Szpigel et al., 2018). The GLP-1R-cAMP pathway also decreases islet inflammation leading to better survival of β-cells (Pugazhenthi et al., 2010), and macrophage infiltration and inflammation of adipose tissue are inhibited by GLP-1 in an obese mouse model of diabetes (Lee et al., 2012). However, the downstream molecular mechanisms are still not clear. We have now identified the role of PKA Cα/TXNIP in the anti-inflammation effects of exendin-4, leading to the β-cell protective effects. This finding provides the missing link between GLP-1R and TXNIP interactions *in vitro*. As the downstream effects of PKA activation can result in various net outcomes, it is still necessary to demonstrate the significance of GLP-1R/PKA/TXNIP signaling pathway in improving the functions of pancreatic β- cells *in vivo*, and this requires needs further study.

In summary, our current study suggested that cAMP/PKA Cα signaling was the major effector pathway in TXNIP degradation within pancreatic β-cells (**Figure 6E**). Clearly, PKA Cα activation enhanced the degradation of TXNIP through phosphorylation, and PKA Cα directly interacted with TXNIP leading to down-regulation of ER stress-induced inflammasome activation in pancreatic β-cells. It is possible that TXNIP is ubiquitinated before degradation, but which E3 ligase participates in this process is still elusive and needs further study. In a word, this study extends our knowledge on PKA Cα/TXNIP signaling in pancreatic β-cells. 

## Data Availability Statement

All datasets generated for this study are included in the manuscript/supplementary files.

## Author Contributions

Participated in research design: SH, WW, and XY. Conducted experiments: SH, WW, YW and CC. Performed data analysis: XT and XY. Wrote or contributed to the writing of the manuscript: KN, SL, and XY.

## Funding

The Natural Science Foundation of China (81603118, 81700854), Pearl River Nova Program of Guangzhou (201806010119), Natural Science Foundation of Guangdong Province (2017A030313717), and “New Drug Creation and Development” major scientific and technological projects of Guangdong Province (2019B020202002) supported this work.

## Conflict of Interest

The authors declare that the research was conducted in the absence of any commercial or financial relationships that could be construed as a potential conflict of interest.

## Abbreviations

ER stress, endoplasmic reticulum; Epac, exchange protein directly activated by cAMP; FSK, forskolin; GLP-1R, glucagon-like peptide-1 receptor; GSIS, glucose-induced insulin secretion; PKA, protein kinase A; TXNIP, thioredoxin-interacting protein; T2D, type 2 diabetes.
